# Antioxidant Activity of Flaxseed Extracts in Lipid Systems

**DOI:** 10.3390/molecules21010017

**Published:** 2015-12-23

**Authors:** Adriana Slavova-Kazakova, Magdalena Karamać, Vessela Kancheva, Ryszard Amarowicz

**Affiliations:** 1 Institute of Organic Chemistry with Centre of Phytochemistry, Bulgarian Academy of Sciences, Acad. G. Bonchev Street, bl. 9, Sofia 1113, Bulgaria; adriana_slawowa@yahoo.com (A.S.-K.); vessy.kancheva@abv.bg (V.K.); 2 Department of Chemical and Physical Properties of Food, Institute of Animal Reproduction and Food Research of the Polish Academy of Sciences, 10-748 Olsztyn, Tuwima 10, Poland; m.karamac@pan.olsztyn.pl

**Keywords:** flaxseed, lignans, antioxidant activity, lipid systems

## Abstract

The aim of this work was to compare the antioxidant activity of the extract of flaxseed and its alkaline hydrolysate in two model systems: lipid autoxidation of triacylglycerols of sunflower oil (TGSO)—in a homogeneous lipid media and during β-carotene-linoleate emulsion system. In addition, pure lignans were tested. The material was defatted with hexane and then phenolic compounds were extracted using dioxane-ethanol (50:50, *v*/*v*) mixture. Carbohydrates were removed from the crude extract using an Amberlite XAD-16 column chromatography. The content of total phenolic compounds in the crude extract and after alkaline hydrolysis was determined using a Folin-Ciocalteu’s phenol reagent. Individual phenolic compounds were determined by nordihydroguaiaretic acid (RP-HPLC) method in gradient system. The alkaline hydrolysis increased the content of total phenolics in the extract approximately by 10%. In the extracts of flaxseed, phenolic compounds were present in the form of macromolecular complex. In the alkaline hydrolysate, secoisolariciresinol diglucoside (SDG) was found as the main phenolic compound. Small amounts of *p-*coumaric and ferulic acids were also determined. SDG and both extracts were not able to inhibit effectively lipid autoxidation. The kinetics of TGSO autoxidation at 80 °C in absence and in presence of the extract before hydrolysis (EBH) and after hydrolysis (EAH) was monitored and compared with known standard antioxidants. Ferulic acid (FA) and butylated hydroxyl toluene (BHT) showed much higher antioxidant efficiency and reactivity than that of both extracts. Secoisolariciresinol (SECO) showed a higher activity in both model systems than SDG. However, the activity of SECO was much lower than that of nordihydroquaiaretic acid (NDGA).

## 1. Introduction

Flaxseed (*Linum usitatissimum* L.) has been the focus of interest of nutritionists due to potential health benefits. It is associated with the presence in the plant of alpha-linolenic acid and lignan secoisolariciresinol diglucoside (SDG) [1]. The seeds of this plant are the richest source of lignans [2]. In human intestinal tract, SDG is converted by bacteria to mammalian lignans: enterodiol (ED) and enterolactone (EL) [3]. Due to the similarity in the chemical structure of ED and EL with oestradiol, both compounds can act as weak estrogenic/antiestrogenic compounds [4].

Flaxseed lignan can reduce the risk of mammary and prostatic tumors, as was confirmed by the results of several studies [5,6,7,8]. The anticancer activity of flaxseed lignans could be caused by its antioxidant properties [1]. Such properties were reported for flaxseed extracts, their fractions and pure SDG [9,10,11,12,13,14,15,16,17,18].

**Figure 1:**
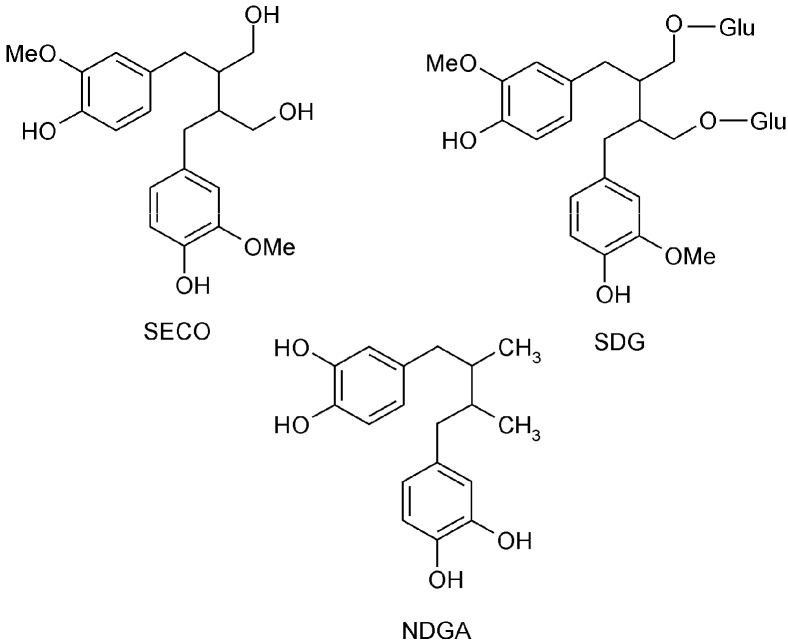
Chemical structure of secoisolariciresinol diglucoside (SDG), secoisolariciresinol (SECO), and nordihydroguaiaretic acid (NDGA).

In flaxseed, SDG is ester-linked to hydroxymethyl-glutaric acid (HMGA), and forms the lignan macromolecule. The other compounds of such structure are *p*-coumaric acid glucoside and ferulic acid glucoside [19,20]. A part of hydroxyl groups in lignan macromolecule is glucosided and therefore they cannot act as oxidation protectors. The hydrolysis can liberate phenolic compounds with the hydroxyl groups from the macromolecule. Nordihydroguaiaretic acid (NDGA) is not a flaxseed lignan but it has been chosen for this study because of the similarity of its main structure with those of SECO and SDG (Figure 1). 

Therefore, the aims of this study are to investigate the effect of chemical hydrolysis of flaxseed extract on its antioxidant activity in various lipid systems and to compare the antioxidant potential of SECO and SDG with that of NDGA.

## 2. Results and Discussion

**Figure 2:**
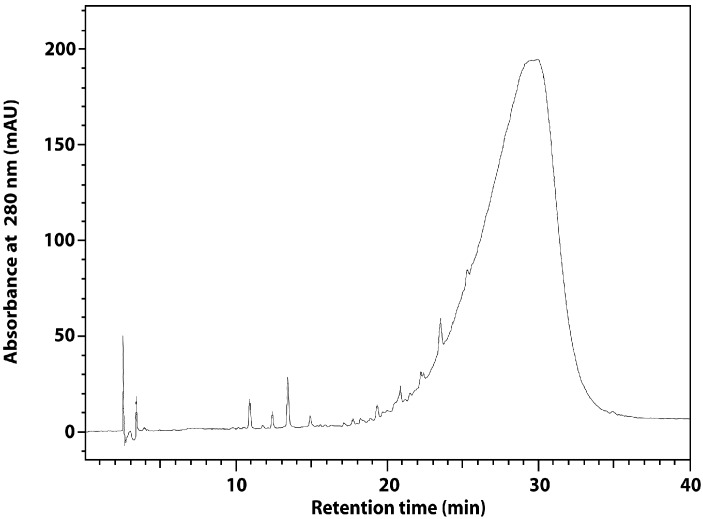
RP-HPLC chromatogram of flaxseed extract before hydrolysis.

**Figure 3:**
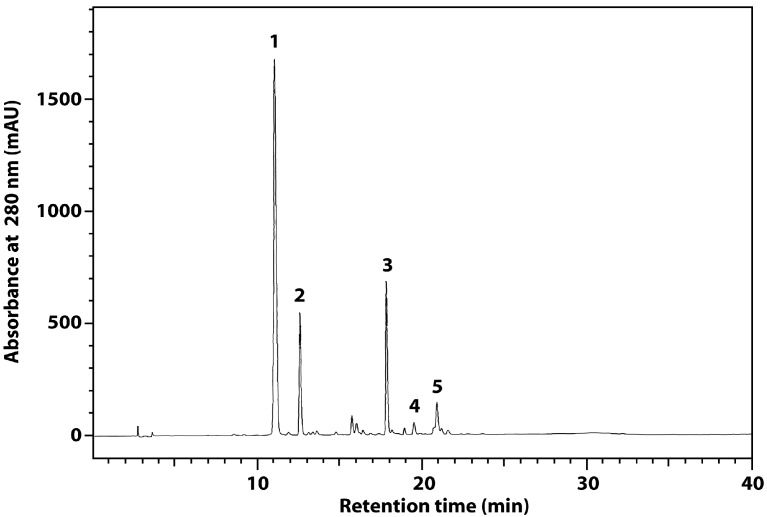
RP-HPLC chromatograms of extract after hydrolysis: (**1**) *p*-coumaric acid glucoside (CoAG); (**2**) ferulic acid glucoside (FeAG); (**3**) SDG; (**4**) *p*-coumaric acid; and (**5**) ferulic acid.

The content of total phenolics in the flaxseed extract is similar to that reported before for rapeseed and canola [21,22] and higher than that for soybean. The alkaline hydrolysis increased the content of total phenolics in the extract by approximately 10% (Table 1). RP-HPLC chromatogram of the flaxseed extract was characterized by broad peak originated from the lignan macromolecule (Figure 2). Such result was also obtained by other authors [19]. The alkaline hydrolysis liberated from the macromolecule SDG and small amounts of *p-*coumaric acid and ferulic acid (Figure 3, Table 1). The results are in line with literature data [23].

**Table 1:** Content of total phenolics and individual phenolic compounds in flaxseed extract and its hydrlolysate.

Material	Total Phenolics (mg CE/g)	SDG (mg/g)	*p*-Coumaric Acid (mg/g)	Ferulic Acid (mg/g)
Extract before hydrolysis	85 ± 5	-	-	-
Extract after hydrolysis	96 ± 5	333 ± 15	3.1 ± 0.2	8.5 ± 0.4

**Figure 4:**
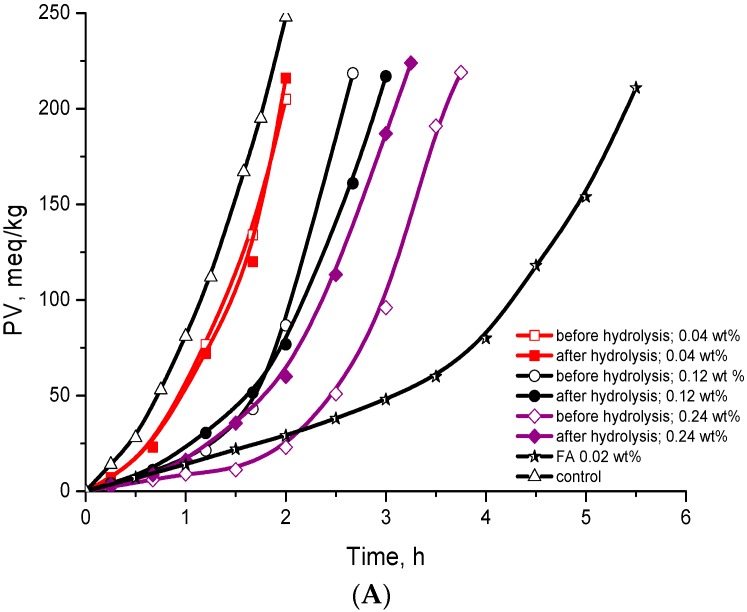

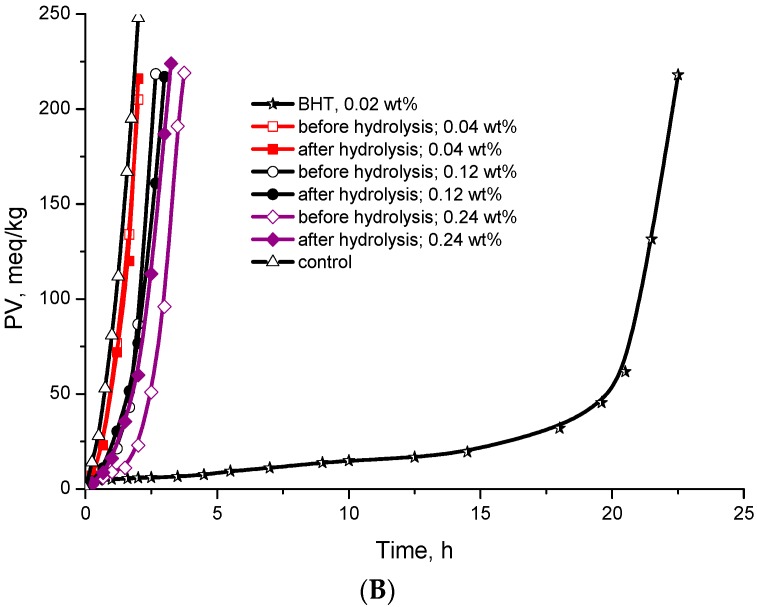
Kinetic curves of lipid hydroperoxides (LOOH) accumulation during oxidation of triacylglycerols of sunflower oil (TGSO) autoxidation at 80 °C in presence of flaxseed extracts before hydrolysis and ferulic acid (FA) (**A**); and in presence of flaxseed after hydrolysis and butylated hydroxyl toluene (BHT) (**B**).

Figure 4 presents the kinetics of triacylglycerols (TGSO) autoxidation at 80 °C in absence (control sample, C) and in presence of the extract before hydrolysis (EBH) and after hydrolysis (EAH) was monitored and compared with known standard antioxidants—ferulic acid (FA) and butylated hydroxyl toluene (BHT). It is seen that both extracts at lower concentrations (0.04 wt % and 0.12 wt %) demonstrated no activity during TGSO bulk phase autoxidation. Only at the highest concentration (0.24 wt %) EBH showed weak antioxidant efficiency (however, not significant differences in activity compared to EAH were detected).

The extract before hydrolysis (EBH) showed a weak increase in its antioxidant efficiency (determined as protection factor (PF)): 1.5-fold higher for 0.12 wt % and two-fold higher for 0.24 wt %, compared with that of 0.04 wt % (see Table 2). The effect of EBH on its antioxidant reactivity (determined as one of the most important kinetic parameter, inhibition degree (ID)) is more significant when the concentration increases (1.9-fold higher for 0.12 wt % and 3.3-fold higher for 0.24 wt %, compared with that for 0.04 wt %, see Table 2).

**Table 2:** Main kinetic parameters characterizing the triacylglycerols of sunflower oil (TSG) autoxidation at 80 °C in presence of following extracts from flaxseed.

Addition	wt %	Antioxidant Efficiency		Antioxidant Reactivity		Activity
		IP_A_ (h)	PF (-)	R_A_ (10^−6^ M/s)	ID (-)	
Extract before hydrolysis	0.04	1.2 ± 0.2 ^a^	1.1	3.9 ± 0.4 ^a^	2.0	No activity
	0.12	1.7 ± 0.2 ^a^	1.5	2.1 ± 0.3 ^a^	3.7	No activity
	0.24	2.5 ± 0.3 ^a^	2.3	1.2 ± 0.2 ^a^	6.5	Weak
Extract after hydrolysis	0.04	1.4 ± 0.2 ^a^	1.3	3.8 ± 0.4 ^a^	2.0	No activity
	0.12	1.8 ± 0.2 ^a^	1.6	2.3 ± 0.3 ^a^	3.4	No activity
	0.24	1.9 ± 0.2 ^a^	1.7	1.8 ± 0.2 ^a^	4.3	No activity
FA	0.02	4.2 ± 0.5	3.2	2.0 ± 0.3	4.4	Moderate
BHT	0.02	20.5 ± 2.5	18.6	0.26 ± 0.06	30.0	Strong

FA: ferulic acid; BHT: butylated hydroxyl toluene; IP_A_: induction periods in presence; PF: protection factor; ID: inhibition degree; (-): dimensionless factor; R_A_: initial rate of lipid autoxidation; Control sample (TGSO kinetically pure): IP_C_ = 1.1 ± 0.2 h; R_C_ = 7.8 ± 0.5 10^−6^, M/s; Values of the same extract addition having different letters differ significantly (*p* < 0.05).

The extract after hydrolysis (EAH) manifested a lower effect with increasing concentrations in comparison with EBH. Its antioxidant efficiency (PF: 1.3 for 0.04 wt %, 1.6 for 0.12 wt % and 1.7 for 0.24 wt %) is almost the same with increasing concentrations of EAH (see Table 2). Much higher effect on its antioxidant reactivity (ID) was obtained, which increases 1.7-fold for 0.12 wt % and 2.2-fold for 0.24 wt % in comparison with ID for 0.04 wt %.

Obviously, there are no differences in antioxidant potential between both extracts (before and after hydrolysis) at lower concentrations (0.04 wt % and 0.12 wt %), *i.e.*, they show the same antioxidant efficiency. However, a weak increase of PF and ID for EBH at the highest concentration (0.24 wt %) was observed.

New order of antioxidant efficiency (as PF) of EBH and EAH at different concentrations was obtained: EBH_24_ (2.3) > EAH_24_ (1.7) ≥ EAH_12_ (1.6) ≥ EBH_12_ (1.5) ≥ EAH_4_ (1.3) ≥ EBH_4_ (1.1) (Table 2).

A similar new order of antioxidant reactivity (as ID) of both extracts was also obtained: EBH_24_ (6.5) > EAH_24_ (4.3) >EBH_12_ (3.7) > EAH_12_ (3.4) > EAH_4_ (2.0) = EBH_4_ (2.0) (Table 2).

Comparison with the known standard antioxidants demonstrated much higher antioxidant efficiency and reactivity of FA and BHT than that of both extracts. The hydroxyl groups present in the lignan macromolecule can protect very weakly lipid oxidation. The benzoic rings in SDG have only one hydroxyl group, which is not sufficient for good protection against lipid oxidation. It seems that after hydrolysis, the antioxidant activity originate mostly from ferulic and *p-*coumaric acids (Table 1). The phenolic OH groups in molecules of CoAG and FeAG are not active because they are glucosided.

**Figure 5:**
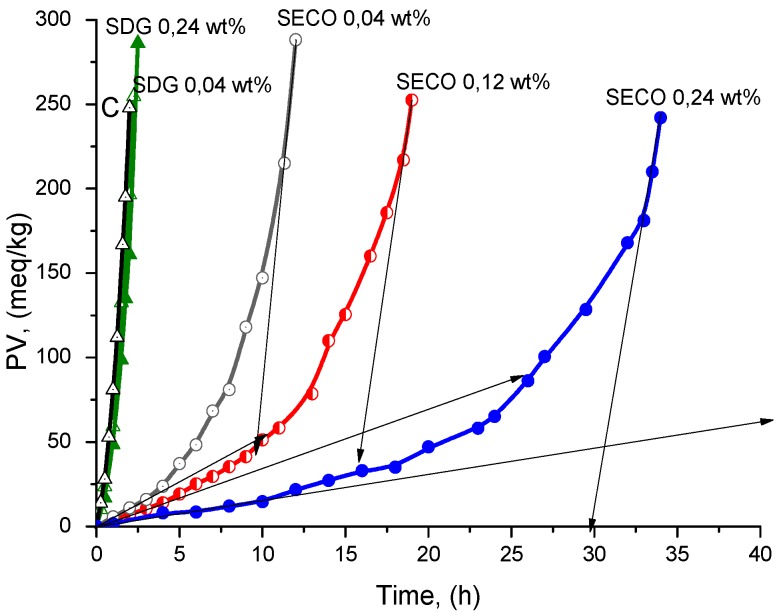
Kinetic curves of LOOH accumulation during the complete TGSO autoxidation process at 80 °C in absence (C/Control) and in presence of flaxseed lignin-secoizolariciresinol (SECO) and secoisolariciresinol diglucoside (SDG).

**Figure 6:**
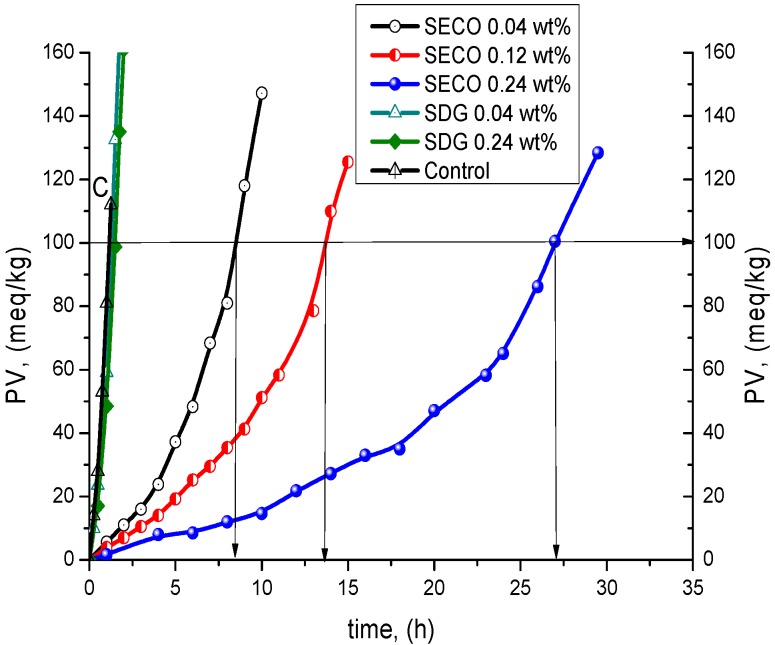
Kinetic curves of LOOH accumulation of TGSO autoxidation process till PV = 100 meq/kg at 80 °C in absence (C, control) and in presence of SECO and SDG.

SECO in a model of lipid autoxidation of triacylglycerols exhibited much better antioxidant activity than SDG (Figure 4 and Figure 5). Comparable kinetic analysis of SECO and SDG demonstrated the following: SECO has 10.4-fold and 37-fold higher antioxidant reactivity (ID) at lower (0.04 wt %) and maximal (0.24 wt %) concentrations used, respectively. At the same time, its antioxidant efficiency (PF) increased with increasing concentrations: 6.7-fold and 18.6-fold (at 0.04 wt % and 0.24 wt %). As can be seen in Figure 5 and Figure 6 and Table 3, SDG in fact did not show any antioxidant potential. This result confirms data reported before [24,25] that the presence of glycoside moiety in the structure of phenolic compounds significantly decreases their antioxidant potential. The latest is due to the possible acceleration of lipid hydroperoxides decomposition from OH groups in glycoside moiety. As a result, the inhibition degree of SDG is much lower because this main kinetic parameter manifests the participation of studied phenolic compounds in side reactions, leading to a decrease of their antioxidant activity [26]. 

NDGA is a much stronger antioxidant than SECO and SDG because of the two catecholic moieties in its structure. For that reason, the comparable kinetic analysis of the three individual phenolic compounds (NDGA, SECO and SDG) was made on the basis of the time to reach peroxide value (PV = 100 meq/kg) according to Gordon [27,28].

**Figure 7:**
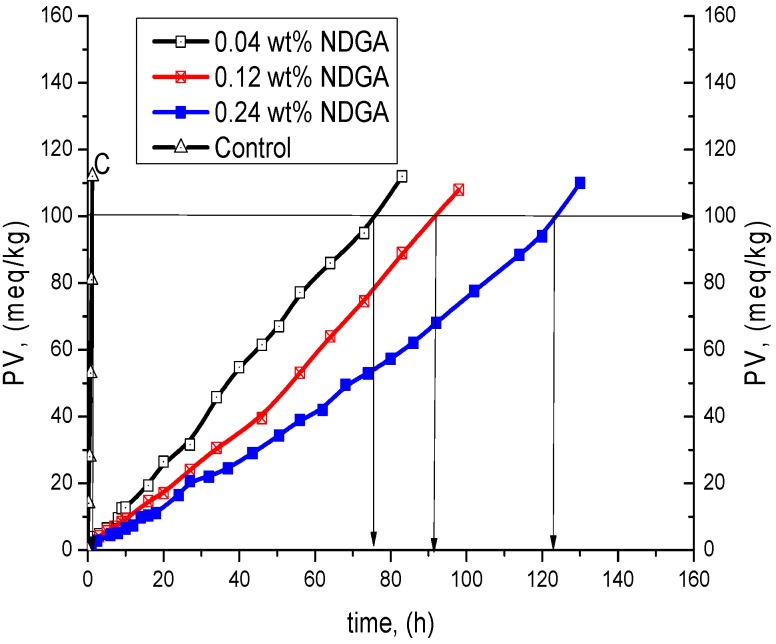
Kinetic curves of LOOH accumulation of TGSO autoxidation process till PV = 100 meq/kg at 80 °C in absence (C, control) and in presence of nordihydroguaiaretic acid (NDGA).

NDGA demonstrated 4.6- to 9-fold higher antioxidant efficiency (PF) and 2.1- to 3.9-fold higher antioxidant reactivity (ID) than that of SECO. At the same time, its efficiency (PF) is 58–80-fold higher and reactivity (ID) is 40–78-fold higher than that of SDG at the same concentrations (Figure 7, Table 3). This fact confirms the importance of catecholic moiety in the structures of phenolic compounds, leading to a regeneration of the antioxidant molecule during TGSO autoxidation by homo-disproportionation reaction of its semiquinone radicals [29].

**Table 3:** Main kinetic parameters, characterizing the TGSO autoxidation process at 80 °C in presence of secoizolariciresinol (SECO), secoisolariciresinol diglucoside (SDG), and nordihydroguaiaretic acid (NDGA).

Compound	wt %	Antioxidant Efficiency		Antioxidant Efficiency at PV = 100 meq/kg		Antioxidant Reactivity	
		IP_A_ (h)	PF (-)	IP_A_^100^ (h)	PF^100^ (-)	R_A_ (10^−7^ M/s)	ID (-)
SDG	0.04	1.4 ± 0.2	1.3	1.3 ± 0.2	1.2	7.8 ± 1.0	1.0
	0.24	1.6 ± 0.2	1.5	1.5 ± 0.2	1.4	7.8 ± 1.0	1.0
SECO	0.04	9.6 ± 0.9	8.7	8.5 ± 0.8	7.7	7.5 ± 0.5	10.4
	0.12	16.1 ± 1.5	14.6	13.7 ± 1.9	12.5	4.9 ± 0.5	15.9
	0.24	30.7 ± 1.9	27.9	27.1 ± 1.7	24.6	2.1 ± 0.3	37.1
NDGA	0.04	-	-	76 ± 4	69	1.9 ± 0.3	41
	0.12	-	-	91 ± 5	8.3	1.3 ± 0.3	60
	0.24	-	-	123 ± 6	112	1.0 ± 0.3	78

IP_A_^100^, PF^100^ – factors calculated for PV = 100 meq/kg; Control sample (TGSO kinetically pure): IP_C_ = (1.1 ± 0.2), h; R_C_ = (7.8 ± 0.5) 10^−6^, M/s.

In the study of Hosseinian *et al.* [13], SECO demonstrated a trend for better protection against oxidative degradation of canola oil than SDG.

**Figure 8:**
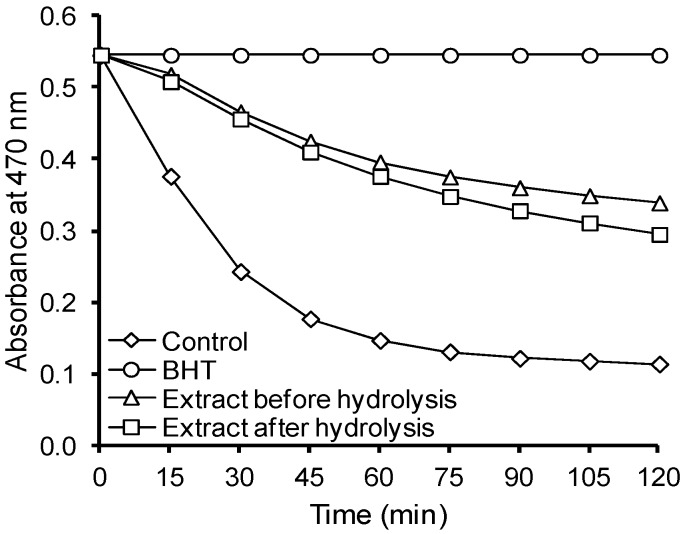
Antioxidant activity of the flaxseed extracts in β-carotene-linoleate emulsion system.

The hydrolysis of the lignan macromolecule slightly decreased its antioxidant activity in emulsion system (Figure 8). The results of β-carotene-linoleate emulsion system were similar to that reported before by Amarowicz *et al*. [9] for flaxseed crude extract and its hydrophobic fraction [10].

**Figure 9:**
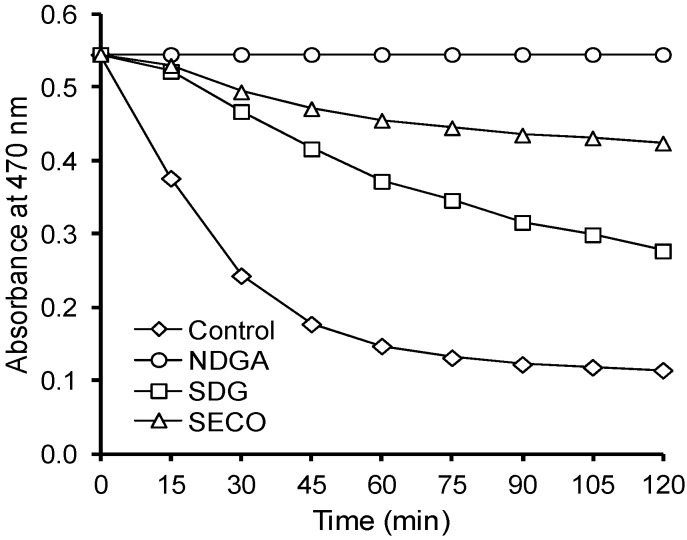
Antioxidant activity of SDG, SECO, and NDGA in β-carotene-linoleate emulsion system.

In emulsion system, SECO exhibited better antioxidant properties than SDG (Figure 9). Similar relation was noted for SECO and SDG before in liposome system [13].

## 3. Experimental Section

### 3.1. Chemicals

All solvents used were of analytical grade unless otherwise specified. Methanol, hexane, and acetonitrile were acquired from the P.O.Ch. Company (Gliwice, Poland). Sephadex LH-20 and Amberlite XAD-16 were obtained from Sigma-Aldrich (Poznań, Poland). RP-18 gel (40–63 μm) was purchased from Merck (Darmstadt, Germany). Standard antioxidants—butylated hydroxyl toluene (BHT) and ferulic acid (FA)—were from Merck (Darmstadt, Germany) and were used without further purification.

### 3.2. Plant Material

Ground, partially defatted flaxseeds were purchased from the “Ekoprodukt” company (Częstochowa, Poland).

### 3.3. Extract Preparation

Phenolic compounds were extracted from 50 g defatted with hexanes flaxseeds using dioxane:ethanol (1:1, *v*/*v*) [17]. The extraction was carried out for 16 h, at 60 °C with continuous shaking in water bath. Then, solvent was evaporated using a Büchi Rotavapor R-200 at 40 °C.

### 3.4. Extract Purification 

The extract of phenolic compounds was purified using column chromatography on Amberlite XAD-16 [23]. A 1.0 g portion of the extract was suspended in distilled water and loaded on the column. Firstly, water-soluble compounds, mainly sugars and low-molecular-weight organic acids, were eluted using distilled water and discarded. The solvent was then changed over to methanol, which eluted the phenolic compounds. The solvent of the collected fraction was removed using Rotavapor.

### 3.5. Extract Alkaline Hydrolysis

The purified extract was subjected to base hydrolysis. Briefly, the purified extract was suspended in 0.3 M NaOH, and left for 2 days at room temperature under continuous stirring. The obtained hydrolysate was acidified to pH 3.0 using 2 M HCl [17] and subjected to column chromatography on RP-18 gel. Water-soluble compounds were eluted with distilled water and discarded, whereas compounds of interest were eluted with methanol. The solvent of the collected fraction was removed using Rotavapor.

### 3.6. Determination of Total Phenolic Content

The content of total phenolics in the extracts was determined using Folin-Ciocalteu’s phenol reagent [22]. The results were expressed as mg catechin equivalent (CE) per g extract. 

### 3.7. RP-HPLC

The extract before and after hydrolysis were analyzed using a Luna C18 (250 × 4.6 mm, 5 μm; Phenomenex, Torrance, CA, USA) column and a Shimadzu system consisting of two LC-10AD pumps, a SCL 10 A system controller, and a SPD-M 10 A diode array detector. A flow rate of 1 mL/min, and gradient elution with acetonitrile:water:acetic acid (5:93:2, *v*/*v*/*v*) (solvent A) and acetonitrile:water:acetic acid (40:58:2, *v*/*v*/*v*) (solvent B), at 0–50 min from 0 to 100% solvent B was employed [30]. The concentration of sample dissolved in methanol was 2 mg/mL, the injection volume was 20 μL; DAD was set at 280 and 320 nm.

### 3.8. SDG and SECO Separation

A fraction rich in SDG was obtained from the hydrolyzed extract using a Sephadex LH-20 column chromatography with methanol as a mobile phase [30]. From this fraction, pure SDG was purified using semi-preparative HPLC on a Luna C18 (250 × 10 mm, 5 μm; Phenomenex) column. A flow rate of 3 mL/min and the same gradient elution as by an analytical HPLC were used. A volume of 500 μL was injected into column. SECO was obtained from SDG after acid hydrolysis (2 M HCl, 2 h, at 100 °C) using applying the same HPLC method.

### 3.9. Preparation of Triacylglycerols

Triacylglycerols of commercially available sunflower oil (TGSO) were cleaned from pro- and anti-oxidants by adsorption chromatography according to Kancheva *et al.* [31] and stored under nitrogen at minus 20 °C. Fatty acid composition of the lipid substrate was determined by GC analysis of the methyl esters of the total fatty acids obtained with a GC-FID Hewlett-Packard 5890 equipment (Hewlett-Packard GmbH, Unterpremstätten, Austria) and a capillary column HP INNOWAX (polyethylene glycol mobile phase, Agilent Technologies, Santa Clara, CA, USA) 30 m × 0.25 mm × 0.25 mm. The temperature gradient started from 165 °C, increased to 230 °C ate 4 °C/min, and held at this temperature for 15 min; injection volume was 1 µL. Injector and detector temperatures were 260 and 280 °C, respectively. Nitrogen was the carrier gas at a flow rate 0.8 mL/min. The analyses were performed in triplicate. Six different fatty acids were present in TGSO: 16:0—6.7%; 18:0—3.6%; 18:1—25.1%; 18:2—63.7%; 20:0—0.2%; and 22:0—0.7%. Lipid samples containing various inhibitors were prepared directly before use. Aliquots of the antioxidant solutions in purified acetone were added to the lipid sample. Solvents were removed under a nitrogen flow.

### 3.10. Lipid Autoxidation

The process was carried out in a thermostatic bath at 80 °C (± 0.2 °C) by blowing air through the samples in special vessels. The oxidation process was monitored by withdrawing samples at measured time intervals and subjecting them to iodometric determination of the primary products (lipid hydroxyperoxides, LOOH) concentration, *i.e.*, the peroxide value (PV) [32]. All kinetic data are expressed as the average of two independent measurements. The results were calculated using the computer programs Origin 6.1 and Microsoft Excel-97.

### 3.11. Kinetic Parameters of the Studied Extracts and Pure Compounds 

For the extracts and pure compounds (SDG, SECO, NDGA, BHT, ferulic acid), the antioxidant efficiency, induction period, protection factor (PF), and inhibition degree (ID) were calculated [26]. Antioxidant efficiency means the potency of antioxidant to increase the oxidation stability of the lipid sample by blocking the radical chain process. It could be presented with induction period. Protection factor (PF) means how many times the antioxidant increases the oxidation stability of the lipid sample and was determined as a ratio between the induction periods in presence (IP_A_) and in absence (IP_C_) of an antioxidant, *i.e.* PF = IP_A_/IP_C_. Inhibition degree (ID) is a measure of the antioxidant reactivity e.g*.*, how many times the antioxidant shortens the oxidation chain length, *i.e.*, ID = R_C_/R_A_, and for that reason is one of the most important kinetic parameters. Initial rates of lipid autoxidation in absence (R_C_) and in presence of antioxidant (R_A_) were found from the tangent at the initial phase of the kinetic curves of hydroperoxides accumulation.

### 3.12. Antioxidant Activity in a β-Carotene-Linoleate Model System

The antioxidant activity of the extracts, SDG, SECO, NDGA, BHT was determined in emulsion system using the method described by Miller [33]. Methanolic solutions (0.2 mL) containing 2 mg of analysed material were added to a series of tubes containing 5 mL of a prepared emulsion of linoleic acid and β-carotene stabilized with Tween 40. Immediately after addition of the emulsion to each tube, the zero-time absorbance at 470 nm was recorded. Samples were kept in a water bath at 50 °C and their absorbance values were recorded over a 120-min period at 15 min intervals.

### 3.13. Statistical Analysis

Results are reported as mean value ± SD. Significance of differences between extract properties before and after the hydrolysis were investigated using Student’s t-test.

## 4. Conclusions

Flaxseed extract, its alkaline hydrolysate and SDG are not able to inhibit effectively lipid autoxidation in TGSO model. Both extracts act as natural antioxidants in a β-carotene-linoleate emulsion system. SECO exhibited a stronger activity than SDG. Due to antioxidant properties in emulsion system, flaxseed extract and its hydrolysate can be used as natural antioxidants for meat, mayonnaise, and dressing, thus prolonging shelf life. 
